# Comprehensive Evaluation of *Bacillus thuringiensis* subsp. *israelensis*: From Molecular Profiling to Ecotope-Specific Larvicidal Efficacy Against Laboratory *Aedes aegypti* and Wild Mosquito Populations

**DOI:** 10.3390/insects17070747

**Published:** 2026-07-22

**Authors:** Mykola Patyka, Renjun Wang, Tetiana Patyka, Anastasiia Honchar, Antonina Kalinichenko

**Affiliations:** 1 Faculty of Life Sciences, Qufu Normal University, Qufu 273165, China; npatyka@gmail.com (M.P.); wangrenjun2002@126.com (R.W.); patykatatyana@gmail.com (T.P.); 2 Department of Phytopathology, National University of Life and Environmental Sciences of Ukraine, 03041 Kyiv, Ukraine; byasya40@gmail.com; 3 Institute of Horticulture, National Academy of Agrarian Sciences of Ukraine, 03027 Kyiv, Ukraine; 4 Institute of Environmental Engineering and Biotechnology, University of Opole, 45-040 Opole, Poland

**Keywords:** *Bacillus thuringiensis* subsp. *israelensis*, mosquitoes, Culicidae, larvicidal properties, δ-endotoxins, ecoengineering, Ukraine

## Abstract

The escalating challenge of managing vector-borne pathologies necessitates the advancement of vector control strategies that are both highly potent and environmentally benign. This research evaluates the insecticidal efficacy and environmental plasticity of three *Bacillus thuringiensis* subsp. *israelensis* (*Bti*) H14 strains 33, 87/1, and 7-1/3 against laboratory and wild mosquito populations. In controlled laboratory assays, cultures with a spore density of 4.0–4.4 × 10^9^ spores/mL demonstrated significant larvicidal activity against larvae of all instars (L_1_–L_4_) of *Aedes aegypti*. The median lethal concentration (LC_50_) was established between 1.15 and 1.48 µL/L, resulting in cumulative mortality rates of 95–96% within a 48 h exposure window. Proteomic profiling via SDS-PAGE and Western blotting validated the robust synthesis of the signature δ-endotoxin complex, specifically verifying the presence of Cry4Aa, Cry4Ba, Cry11Aa, and Cyt1Aa. Field applications in specific forest (Polissya) and steppe (Forest–Steppe) aquatic biotopes of Ukraine—characterized by distinct physicochemical profiles (differing significantly in light exposure, water hardness, salinity, and suspended solids)—substantiated the stability of the liquid formulations, achieving larval suppression rates of 95–100% within 48 h against wild *Aedes*-dominated assemblages at application rates of 0.25–1.0 mL/m^2^ (equivalent to 2.5–10.0 L/ha). The demonstrated environmental adaptability suggests these novel strains are viable candidates for broad-spectrum ecological engineering applications. Their integration into sustainable vector control strategies offers a mechanism to enhance biosecurity without compromising the ecological balance of local biocenoses.

## Simple Summary

This study provides a comprehensive evaluation of three novel autochthonous *Bacillus thuringiensis* subsp. *israelensis* (*Bti* 33, 87/1, and 7-1/3) strains from Ukrainian ecosystems for mosquito biocontrol. Molecular analysis confirmed the stable synthesis of specific insecticidal delta-endotoxins (Cry4Aa, Cry4Ba, Cry11Aa, and Cyt1Aa), ensuring targeted larvicidal efficacy against *Aedes aegypti* larvae in laboratory assays and wild mosquito populations under field conditions. The study demonstrated high environmental stability of the liquid *Bti*-based formulations and highlighted the biotechnological potential of these native isolates for the production of sustainable biolarvicides.

## 1. Introduction

The global expansion of *Aedes aegypti* continues to pose a significant threat to public health as the primary vector for Dengue, Zika, and Yellow fever. Despite the availability of synthetic insecticides, the rapid development of resistance in mosquito populations and the growing environmental concerns necessitate the implementation of biological control strategies. Among these, *Bacillus thuringiensis* subsp. *israelensis* (*Bti*) remains the most effective and environmentally safe bio-larvicide due to its highly specific synergistic δ-endotoxins [1,2].

However, the efficacy of *Bti*-based products in natural aquatic ecosystems is often limited by environmental factors such as solar radiation, organic matter content, and temperature. This underscores the importance of identifying and characterizing native *Bti* strains that exhibit high larvicidal activity and environmental stability within specific regional ecotopes [3,4]. In Ukraine, the search for autochthonous strains with unique toxicological profiles remains a priority for developing localized biotechnological solutions for mosquito control.

Consequently, the paradigm of vector control has shifted towards ecological engineering, prioritizing microbial agents such as *Bacillus thuringiensis* (*Bt*). These biopesticides offer a cost-effective alternative that mitigates chemical accumulation in ecosystems, delays resistance development, and ensures biosafety for humans and non-target fauna. Developing robust biological control strategies requires a deep understanding of the vector microbiome and its metabolic potential [5].

Bacterial biocontrol is currently a cornerstone of mosquito management. This approach utilizes entomopathogenic bacteria, including *B. thuringiensis* as a larvicide [6,7], and *Wolbachia* spp. to inhibit vector competence and development. Microorganisms within the genera *Bacillus*, *Brevibacillus*, and *Lysinibacillus* offer valuable resources for controlling vectors such as *Aedes*, *Anopheles*, and *Culex* [8]. These bacteria synthesize a repertoire of virulence factors and toxic molecules—including Cry, Cyt, Vip, Mpp, Tpp, and CpbB proteins, as well as chitinases—which are integral to contemporary biological control programs [9].

*Bt* is distinguished from related species, such as *B. cereus* and *B. anthracis*, by its capacity to produce crystalline parasporal inclusions during sporulation [10]. These inclusions, comprising Cry and Cyt proteins, demonstrate potent toxicity primarily against dipteran targets (Culicidae, Simuliidae, Chironomidae). While highly selective, the ecological impact and potential non-target effects of *Bti* on other functionally related or distant aquatic arthropod groups continue to be the subject of comprehensive ecological monitoring [11,12,13].

Urbanization and environmental shifts—including changes in hydrological regimes, sanitation, and climate—have driven significant increases in mosquito density [14]. Anthropogenic pollution often creates optimal breeding grounds. Urban environments have influenced mosquito evolution, notably in the differentiation of *Culex pipiens* in surface waters and the *Culex molestus* ecotype, which colonizes underground and flooded biotopes [15].

The diversity, phenology, and ecological distribution of mosquitoes in Europe necessitate the development of region-specific, integrated control programs. The present study focuses on evaluating novel bacterial strains as agents of ecological engineering, offering a targeted mosquito control solution that functions within aquatic ecosystems with minimal disruption.

In this context, three novel isolates of *Bti* H_14_—designated as strains 33, 87/1, and 7-1/3—were recovered from natural biotopes. These strains are notable for their high spore productivity and potent larvicidal activity. Their significance lies in their isolation from specific geographical zones and their consistent efficacy across diverse ecological conditions, suggesting unique adaptive traits. Preliminary screening indicates that these isolates may outperform existing analogues in terms of stability and speed of action, positioning them as promising candidates for next-generation biolarvicides.

The specificity of *B. thuringiensis* pathogens is dictated by the biochemical structure of the endotoxins and their interaction with the target insect. The genetic diversity of these toxins is evolutionarily significant, ensuring the survival and adaptability of the bacterium in various biocenoses. Currently, approximately 500 distinct δ-endotoxins have been identified [6,9]. The larvicidal efficacy of *Bti* is primarily driven by four major polypeptides (134, 128, 72, and 27 kDa) and at least two minor ones (78 and 29 kDa), encoded by the genes cry4Aa, cry4Ba, cry11Aa, cyt1Aa, cry10Aa, and cyt2Ba. These genes are located on the pBtoxis plasmid [16].

Formulations combining *Bti* and *B. sphaericus* (*Bs*) leverage the strengths of both bacteria, providing efficacy in both clear and organic-rich waters and extending persistence up to 28 days. Moreover, these combinations often exhibit synergism, enhancing toxicity against vectors like *Culex quinquefasciatus* and *Aedes aegypti*. As previously discussed regarding the evolutionary potential of endospore-forming bacteria, this synergistic interaction serves as a robust mechanism against resistance development while maintaining selective larvicidal action [17]. The mode of action, particularly for Cry11Aa, is complex and likely involves mechanisms extending beyond simple pore formation.

The systematic screening for potent autochthonous *Bti* strains remains a cornerstone of modern bio-insecticide development [18]. Globally, researchers emphasize that native isolates often possess unique genetic variations and physiological adaptations to local environmental conditions, providing higher persistence and toxicity compared to standardized commercial strains [19]. For instance, extensive screening programs in Brazil, India, and Southeast Asia have successfully identified highly active strains through a combination of traditional bioassays and molecular fingerprinting of cry and cyt gene clusters [20,21]. Recent studies highlight that the discovery of new local isolates not only contributes to resistance management strategies but also reduces the costs associated with the importation of biopesticides. Such screening efforts typically involve a multi-tiered approach: from primary toxicity testing against diverse mosquito instars to the detailed characterization of the δ-endotoxin crystalline inclusions, ensuring the selection of candidates with the highest biotechnological potential [7].

The objective of this research was to conduct a comprehensive integrated evaluation of three autochthonous *Bti* strains (33, 87/1, and 7-1/3) isolated from Ukrainian ecosystems. The study focused on correlating their molecular–proteomic profiles (specifically the expression of Cry and Cyt δ-endotoxins) with larvicidal efficacy against *Aedes aegypti* under both standardized laboratory conditions and diverse natural aquatic ecotopes of the Forest and Steppe zones. This integrated approach by combining molecular identification of protein toxins with functional laboratory assays and field trials in diverse environmental matrices to provide a complete evaluation of the technological potential of the novel *Bti* strains with environmental plasticity for future biotechnological applications.

## 2. Materials and Methods

### 2.1. Biotesting

To evaluate larvicidal efficacy, a stable population of *Aedes aegypti* (*Ae. aegypti*) was used, which meets the international standards established by the Pasteur Institute for biological assays [22]. The selection of *Ae. aegypti* for laboratory bioassays was deliberate, as this species serves as the internationally recognized reference model for standardized toxicological benchmarking of *Bti* formulations according to World Health Organization (WHO) guidelines. Utilizing this standard insectary strain allows for the precise determination of baseline larvicidal potency and facilitates direct comparisons of the novel autochthonous isolates with globally documented *Bti* strains, prior to evaluating their ecological plasticity against native mosquito assemblages in field trials. In the laboratory models, 3rd and 4th instar larvae obtained from a genetically homogeneous population were used. The colony was maintained under controlled, pathogen-free conditions in an insectarium, with environmental parameters set at a temperature range of 28–30 °C, a relative humidity of 60% to 80%, and a photoperiod of 12 h light/12 h dark.

**Figure 1:**
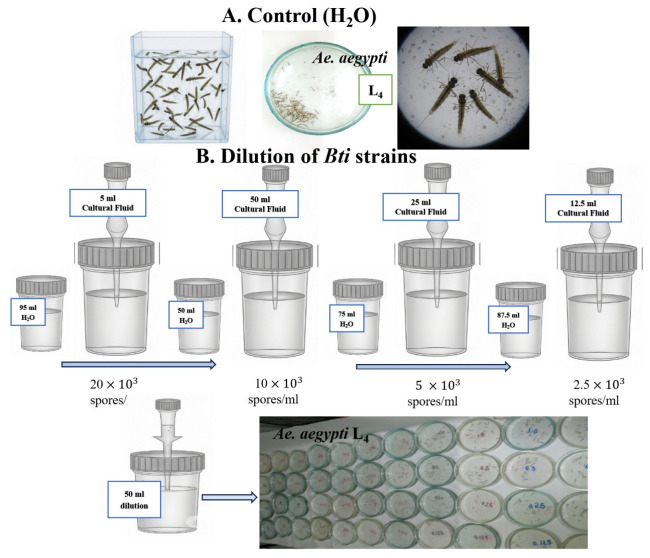
Testing of larvicidal activity of strains *Bti* (insect population *Ae. aegypti*).

Larval rearing was conducted in cuvettes containing distilled water filled to a depth of 2 cm, maintained at a constant temperature of 28 °C with a 12 h light cycle. The dietary regime consisted of a dry defatted yeast mixture provided until pupation. The complete life cycle, including development to the imago stage and oviposition on filter substrates, was supported in specialized insectary cages. For experimental synchronization, larvae hatching within a tight 1–2 h window were selected and reared in water cuvettes for 4 days to reach the 3rd instar stage, as depicted in the experimental design (Figure 1).

Figure 1 illustrates the comprehensive testing scheme for assessing the functional activity of *Bti* H_14_ strains against the *Ae. aegypti* insectary population, detailing both the aqueous control (H_2_O) and the preparation of strain suspension dilutions. Complementary studies involved wild mosquito populations (Diptera: Culicidae) sampled directly from natural, primarily aedogenic, aquatic biotopes.

### 2.2. Bacterial Strains Isolation and Maintenance

The native *Bti* (H_14_) strains were isolated from soil and water samples collected in the Forest and Steppe zones of Ukraine. The isolation was performed using the selective heat-shock method (80 °C for 15 min) to eliminate non-spore-forming microbiota, followed by plating on nutrient agar (NA). Pure cultures were identified by the presence of parasporal crystalline inclusions under phase-contrast microscopy. For long-term storage, the strains were maintained as 20% glycerol stocks at −80 °C and in a lyophilized state. Working cultures were kept on NA slants at 4 °C with subculturing every 3 months.

### 2.3. Taxonomic Identification

The taxonomic affiliation of the isolates was confirmed through a polyphasic approach. Morphological characterization included Gram staining, assessment of colony morphology, and observation of rhomboidal parasporal crystals characteristic of the *israelensis* subspecies. Biochemical profiling was performed according to the established protocols [23]. The following tests were conducted: catalase activity, starch hydrolysis, Voges–Proskauer test (acetoin production), and citrate utilization. The H-serotyping of the flagellar antigens was conducted using the standard agglutination test with specific H_14_ antisera provided by the (D.K. Zabolotny Institute of Microbiology and Virology of the National Academy of Sciences of Ukraine, Kyiv, Ukraine), following the methodology described by de Barjac. Briefly, bacterial cultures were grown to the logarithmic phase to ensure high motility, and flagellar antigens were reacted against a series of H-antisera. A strong positive agglutination reaction specifically with the H_14_ antiserum confirmed the isolates as *Bacillus thuringiensis* subsp. *israelensis*. The specific serotype H_14_ was further validated by the characteristic δ-endotoxin profile (Cry4, Cry11, and Cyt1 proteins) visualized via SDS-PAGE analysis.

### 2.4. Cultivation and Preparation

Fermentation was conducted using submerged culture techniques in Luria–Bertani (LB) broth within Erlenmeyer flasks. The medium composition included peptone, yeast extract, and sodium chloride in a 1:0.5:1 ratio (*w*/*v*) with distilled water adjusted to pH 7.2. Cultures were incubated on thermostatic orbital shakers at 220 rpm and 28–30 ± 1 °C for a duration of 72 h, a period sufficient to achieve 80–90% sporulation and significant release of spore–crystal complexes. The inoculum volume was standardized to at least 4.0% of the total medium volume (50 or 100 mL).

### 2.5. Preparation of the Liquid Formulation for Field Trials

The liquid formulation tested in the field ecotopes was prepared using the whole culture broth obtained after 72 h of submerged fermentation. Following the completion of the fermentation process, the broth—containing a mixture of vegetative cells, spores, and parasporal crystalline endotoxins—was subjected to standardization and stabilization.

The formulation process involved the following steps: 1. Standardization: The concentration of viable spores was adjusted to a titer of 4.0 × 10^9^ CFU/mL (colony-forming units) by dilution with sterile distilled water or concentration via low-speed centrifugation (4000 rpm), depending on the initial batch density. 2. Stabilization: To enhance the shelf-life and environmental persistence of the toxins, glycerol 5% (*v*/*v*) was added as a stabilizer and anti-evaporant. 3. Preservation: Sodium benzoate 0.1% (*w*/*v*) was added as a preservative to prevent microbial contamination. 4. pH Adjustment: The final pH of the liquid formulation was adjusted to 6.5–7.0 using 0.1 M HCl to maintain the integrity of the δ-endotoxin crystals. This standardized liquid technical concentrate was used for all field applications to ensure a uniform dosage of the active insecticidal components across different aquatic biotopes. The final liquid formulation consisted of a standardized technical concentrate: 4.0 × 10^9^ CFU/mL of spore–crystal complex, 5% (*v*/*v*) glycerol, and 0.1% (*w*/*v*) sodium benzoate. To ensure consistency, all field efficacy assessments were performed at standardized intervals of 24, 48, and 72 h post-application.

### 2.6. Quantitative Analysis and Bioassay Protocols

Bacterial density was quantified via conventional plate counting techniques on agar media, ensuring a titer of at least 1.5–2.0 × 10^9^ CFU/mL of culture fluid. Morphological monitoring of the spore-forming cultures was performed using an EVOS FL (Thermo Fisher Scientific, Bothell, WA, USA) digital imaging system. For bioassays, aqueous suspensions were prepared in chlorine-free water using a two-fold serial dilution series (1:200,000 to 1:1,600,000), corresponding to a gradient of nominal bacterial concentrations: 0.5, 0.25, 0.125, and 0.063 × 10^−3^% (*v*/*v*) of the stock suspension. Experimental setups involved placing 25 mosquito larvae into Petri dishes containing 50 mL of the respective suspension dilution, with four replicates per concentration. Nutritional support was provided by adding 0.1–0.2 mL of a 5.0% aqueous dry yeast suspension. The samples were incubated at 28–30 °C, with mortality assessments conducted at 24 and 72 h. Tap water served as the negative control, while the commercial biolarvicide Bactoculicide (Enzim Biotech, Ladyzhyn, Ukraine) was utilized as the positive control in all laboratory experiments to verify larval susceptibility and provide a baseline for efficacy comparison. This reference formulation is based on *Bti* (serotype H_14_) strain 7-1/23, characterized by a viable spore titer of at least 3.5 × 10^9^ CFU/mL and a standardized biological potency rating of 1800 ITU/mg.

All biological assays were performed at an ambient temperature of 25–28 °C. The primary metric for functional activity was the median lethal concentration (LC_50_), defined as the concentration inducing 50% mortality following free ingestion of the spore–crystal complex. Testing protocols adhered to the guidelines established by the World Health Organization for larvicides [24]. Since natural mortality in the negative control groups remained at 0% throughout the entire exposure period, mathematical correction of the mortality data was not required, and raw bioassay counts were directly utilized for the downstream binomial GLM modeling framework. The LC_50_ values and their corresponding 95% confidence intervals were systematically calculated within the binomial GLM framework utilizing the probit link function in R, as detailed in Section 2.9.

### 2.7. Field Trial Methodology

Field efficacy trials were conducted in two distinct agro-climatic macro-zones of Ukraine: the Polissya region and the Forest–Steppe region, targeting wild mosquito populations. To ensure the strict independence of observations and structural replication, a randomized complete block design (RCBD) was implemented. For each region, isolated temporary water bodies and artificial reservoirs (standardized plastic containers, 50 L volume, 0.5 m^2^ water surface area, filled with local biotope water containing baseline organic matter) were selected and established as independent experimental units. These macro-zones exhibited distinct physicochemical profiles: ethe Polissya forest biotopes served as shaded, lower-hardness profiles with lower salinity, whereas the Forest–Steppe sites represented open, sun-exposed profiles characterized by elevated water hardness, higher salinity, and increased total suspended solids (TSS).

For each of the three novel *Bti* strains (33, 87/1, and 7-1/3) and the control (Bactoculicide), four independent replicates (*n* = 4 experimental reservoirs per treatment) were allocated. To prevent chemical cross-contamination, physical disruption, or biological drift during application, all experimental and control reservoirs were spatially segregated, maintaining a minimum buffer distance of 15 m from one another.

The liquid formulations were applied at area-specific dosages of 0.25, 0.5, and 1.0 mL/m^2^ (equivalent to 2.5, 5.0, and 10.0 L/ha, respectively) using manual spraying equipment calibrated to ensure uniform surface distribution. Untreated reservoirs within the identical ecological context, situated upwind from the treated groups, served as negative controls to guarantee that natural fluctuations in the larval populations were accurately documented. Larval density assessments were performed using a standard 350 mL dipper sampling technique immediately prior to application (baseline) and at 24 and 48 h post-treatment.

A rigorous morphological identification protocol was implemented to confirm the taxonomic architecture and genus/species composition of wild mosquitoes both before and after treatment. At baseline (0 h) and after treatment (24 and 48 h), random subsamples of larvae (n=100 individuals per experimental tank or all surviving individuals if density fell below this threshold) were collected using a standard 350 mL dipper and immediately fixed in situ in 70% ethanol to preserve diagnostic structures. Laboratory taxonomic screening was limited to fourth-instar larvae ($L_{4}$) and was performed using binocular stereomicroscopy. Identification was based on key stable diagnostic features, including siphon configuration and siphonal index, number, shape, and location of comb scales, structure and attachment of the siphonal tuft, and branching patterns of frontal, parietal, and postclypeal head hairs. To ensure diagnostic accuracy, the taxonomic analysis was performed independently in duplicate according to the standard morphological keys [25,26]. This allowed for the characterization of the naturalized field populations, which were identified as mixed Culicidae assemblages with a clear dominance of floodwater *Aedes* species and co-occurring populations of the *Culex pipiens* complex.

### 2.8. Molecular-Biological Analysis of Bti H_14_ Crystalline δ-Endotoxins

#### 2.8.1. Protein Extraction and Preparation

Following a 72 h cultivation period required to reach technological maturity and complete sporulation, the *Bti* H_14_ cultures (strains 33, 87/1, and 7-1/3) were harvested to isolate the spore–crystal complexes. Separation was achieved via centrifugation at 10,000 *g* for 15 min at 4 °C. The resulting pellet was resuspended in a lysis buffer composed of 50 mM Tris-HCl (pH 8.0), 150 mM NaCl, 1% Triton X-100, and a 1x protease inhibitor cocktail. Cellular disruption and the subsequent release of crystalline proteins were facilitated by sonication (three 30 s cycles at 50% amplitude). The lysate was then clarified by centrifugation at 12,000× *g* for 10 min at 4 °C, and the supernatant containing the solubilized toxins was retained for downstream analysis.

#### 2.8.2. Electrophoretic Analysis (SDS-PAGE)

Total protein concentration was quantified utilizing the Bradford assay [27]. Aliquots containing 20 µg of protein per lane were resolved by sodium dodecyl sulfate–polyacrylamide gel electrophoresis (SDS-PAGE) using a 10% separating gel. Post-electrophoresis, protein bands were visualized by staining with Coomassie Brilliant Blue R-250, and the resulting protein profiles were digitally documented. Molecular weights were estimated by comparison against a standard marker (Thermo Scientific PageRuler Prestained Protein Ladder). The original, uncropped, and unadjusted full-length gel image is available as Figure S1 in the Supplementary Materials.

#### 2.8.3. Western Blotting

To validate the expression of specific *Bti* toxins, immunoblotting was performed. Proteins separated by SDS-PAGE were transferred onto a 0.45 µm nitrocellulose membrane using a semi-dry transfer system. Non-specific binding sites were blocked by incubating the membrane in 5% skim milk dissolved in TBST (Tris-buffered saline supplemented with 0.1% Tween 20) for one hour at ambient temperature. The membranes were then probed overnight at 4 °C with specific rabbit polyclonal primary antibodies targeting Cry4Aa, Cry4Ba, Cry11Aa, and Cyt1Aa, which were generously provided by the Zabolotny Institute of Microbiology and Virology of the National Academy of Sciences of Ukraine (Kyiv, Ukraine). These antibodies were used at a working dilution of 1:2000. The specificity of the antiserum was previously validated via immunoelectrophoresis and cross-reactivity testing against purified delta-endotoxins of *Bacillus thuringiensis*, ensuring distinct recognition of each target protein without non-specific background binding. Following a wash step with TBST, the blots were incubated for one hour at room temperature with horseradish peroxidase (HRP)-conjugated goat anti-rabbit IgG secondary antibodies (Abcam, Cat ab6721, diluted 1:5000). Protein detection was achieved using an enhanced chemiluminescence (ECL) substrate (Pierce) and imaged via the ChemiDoc XRS+ system (Bio-Rad Laboratories, Inc., Hercules, CA, USA). The full-length, unedited raw membrane images for all replicates are provided in the Supplementary Materials (Figures S2–S5).

### 2.9. Statistical Analysis

The statistical framework was implemented using Generalized Linear Models (GLMs) to account for the proportional and inherently binomial nature of the larval mortality data. For laboratory bioassays, the bivariate response variable was modeled as a matrix of the number of dead and surviving larvae for each replicate using a binomial error distribution family. The logit link function was applied for the factor interaction analysis, while a probit link function was utilized to derive the median lethal concentrations (LC_50_) and their associated 95% confidence intervals (CIs) according to the classic probit analysis framework [28]. The laboratory GLMs evaluated the fixed effects of the distinct bacterial strains (and their formulations), suspension concentrations, exposure durations (24 and 48 h), and their full multi-way interactions (Strain × Concentration × Time). For field trials, the analytical framework was extended to Generalized Linear Mixed Models (GLMMs), where biological zones/biotopes (Polissya and Forest–Steppe) were treated as random factors to account for spatial dependencies and ensure the independence of observations.

Residual diagnostics, including rigorous evaluations for overdispersion, zero-inflation, and systematic deviations from the binomial distribution, were performed using simulated randomized quantile residuals via the DHARMa package (version 0.4.7). In cases where significant overdispersion was detected, a quasibinomial distribution family was implemented. Post hoc pairwise comparisons of estimated marginal means (EMMeans) among treatment groups and commercial controls were conducted using Tukey’s Honestly Significant Difference (HSD) test adjustment at a significance threshold of *p* < 0.05. All statistical modeling, dose–response evaluations, and diagnostic procedures were executed within the R statistical environment (v4.3.2). Graphical representations display modeled means with error bars representing the standard error (SE) derived from the model coefficients, with statistical significance indicated by distinct lettering. Fully reproducible R Markdown scripts (.Rmd) containing data simulation matrices and comprehensive model outputs are provided as Supplementary Materials Files S1 and S2.

Quantitative densitometric data derived from the proteomic expression profiles (SDS-PAGE and Western blot analyses) were evaluated as continuous variables. The statistical significance of differences in the relative expression levels of specific δ-endotoxins (Cry and Cyt proteins) among the three native *Bti* H_14_ strains was determined using a One-way Analysis of Variance (ANOVA). Following ANOVA, post hoc multiple comparisons were conducted using Tukey’s Honestly Significant Difference (HSD) test. The significance threshold was established at *p* < 0.05. Densitometric quantification was performed using ImageJ software (version 1.54g; National Institutes of Health, Bethesda, MD, USA), and the corresponding statistical evaluations were executed within the R statistical environment.

## 3. Results

### 3.1. Molecular and Proteomic Characterization of Native Bti Strains

**Figure 2:**
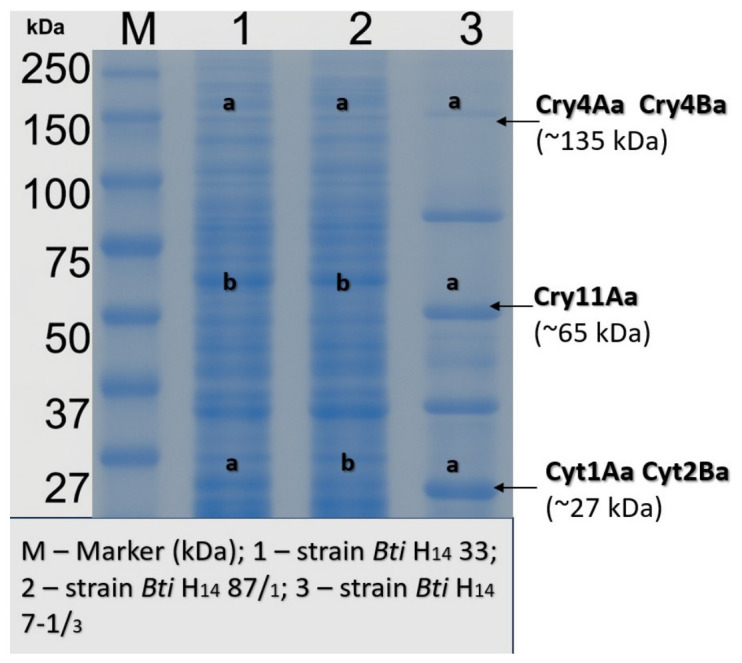
Electrophoretic characterization (SDS-PAGE) of the crystalline δ-endotoxin complex produced by the *Bti* H_14_ strains 33, 87/1, and 7-1/3. The gel image shows a representative profile. Statistical annotations (a, b) assigned to specific protein bands indicate significant differences in their relative densitometric expression levels (based on the mean of three independent replicates) according to One-way ANOVA followed by Tukey’s HSD test (*p* < 0.05).

Initial characterization of the three native isolates focused on their protein profiles to confirm the presence of key δ-endotoxins. The proteomic characterization of the crystalline inclusions recovered from strains 33, 87/1, and 7-1/3 corroborated the existence of a typical delta-endotoxin complex associated with *Bacillus thuringiensis* subsp. *israelensis*. Electrophoretic separation via SDS-PAGE (Figure 2) revealed distinct banding patterns consistent with the known molecular masses of *Bti* Cry and Cyt proteins. Electrophoretic analysis confirmed that all three *Bti* H_14_ strains possessed the characteristic endotoxin polypeptide profile (see Figure S1 for the original uncropped gel).

Examination of the electropherograms for each isolate highlighted prominent bands positioned at approximately 135 kDa, 128 kDa, 65 kDa, and 27 kDa; these signals were attributed to Cry4Aa, Cry4Ba, Cry11Aa, and Cyt1Aa, respectively. Furthermore, the analysis detected lower-intensity bands indicative of minor polypeptide components, thereby confirming the expression of the major larvicidal polypeptide complex characteristic of serotype H_14_. The high degree of uniformity observed in the protein profiles across the three novel strains suggests a conserved genetic basis for the synthesis of these primary larvicidal agents.

**Figure 3:**
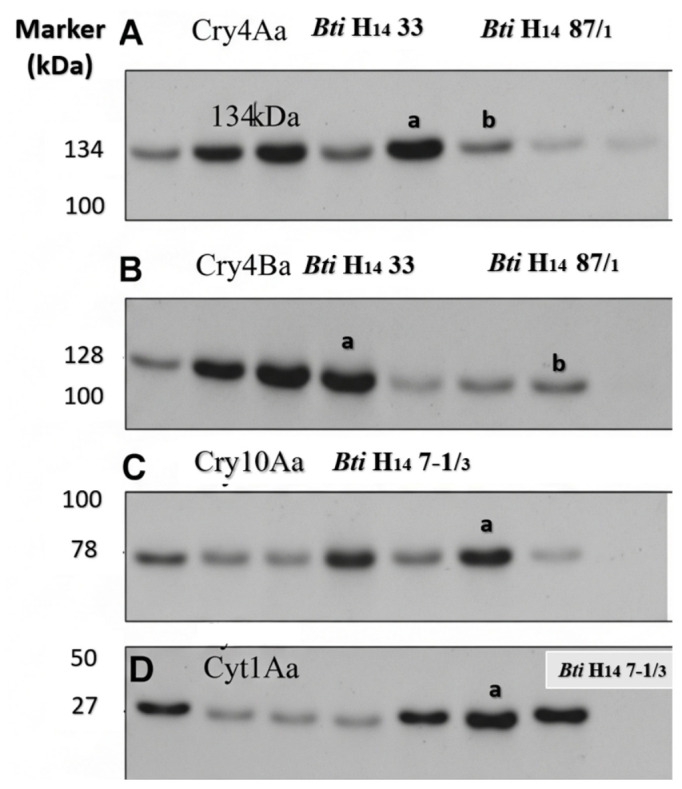
Western blot analysis for the identification of Cry and Cyt δ-endotoxins in *Bti* H_14_ strains: (**A**) Detection of Cry4Aa, (**B**) Cry4Ba, (**C**) Cry10Aa, and (**D**) Cyt1Aa. The panels display representative immunoblots. Statistical annotations (a, b) above the bands indicate significant differences in relative protein expression levels determined by densitometric analysis (based on the mean of three independent replicates) according to One-way ANOVA followed by Tukey’s HSD test (*p* < 0.05).

Subsequent Western blot analysis (Figure 3), utilizing specific antibodies raised against the principal *Bti* toxins, served to verify both the identity and the high-level expression of these proteins within the studied isolates. Intense immunoreactivity was detected at the anticipated molecular weights, offering conclusive evidence that the proteins visualized are indeed functional Cry and Cyt toxins. The comprehensive set of full-length, unedited raw Western blot membrane images for all biological replicates is available in the Supplementary Materials (Figures S2–S5).

Densitometric analysis of the Cry and Cyt protein bands in Figure 2 and Figure 3 was performed using ImageJ software (National Institutes of Health, USA) to evaluate the relative expression levels across the three native *Bti* H_14_ strains. The optical density of each specific band was quantified and normalized to ensure accurate comparison. Statistical significance of the differences in protein expression between isolates was determined using One-way ANOVA followed by Tukey’s HSD test (*p* < 0.05), with distinct letters on the figures indicating significant differences in toxin production levels.

### 3.2. Laboratory Larvicidal Potency Against Aedes aegypti

The experimental evaluation of *Bti* H_14_ strains—specifically isolates 33, 87/1, and 7-1/3—demonstrated robust spore production capabilities alongside potent larvicidal effects. Analysis of the culture fluid, characterized by a spore concentration reaching 4.0 × 10^9^ spores yield/mL of culture fluid, determined that the median lethal concentrations (LC_50_) for fourth-instar *Ae. aegypti* ranged from 1.15 to 1.48 µL/L (Table 1). These metrics align with established efficacy benchmarks, thereby validating the high functional toxicity of these novel H_14_ serotype isolates.

Comparative analysis of the in vitro larvicidal activity (Table 1) revealed that native strains 33 and 87/1 exhibited the highest absolute toxicity against fourth-instar (L_4_) larvae among the novel isolates, with LC_50_ values of 1.25 and 1.33 µL/L, respectively. While strain 7-1/3 displayed a slightly higher LC_50_ (1.48 µL/L) in laboratory conditions—indicating marginally lower baseline toxicity compared to strains 33 and 87/1—it was selected as a priority candidate due to its exceptional ecological plasticity and sustained efficacy in complex, organic-rich field ecotopes (as further demonstrated in field trials). This specific environmental resilience establishes strain 7-1/3 as a highly competitive base for new biotechnological formulations.

The investigated *Bti* H_14_ strains demonstrated rapid insecticidal action, yielding high efficacy rates within a 24 to 48 h observation window. Because the pathogenicity of *Bti*, similar to other *B. thuringiensis* varieties, is contingent upon ingestion, its toxic effects are maximally realized during the vigorous feeding phases of larval development. Consequently, significant mortality among *Ae. aegypti* fourth-instar (L_4_) larvae was recorded within the first day of treatment (Table 2). Conversely, the negative control groups, which were maintained in pathogen-free water, exhibited survival rates of 100%. The mechanism of lethality involves the ingestion of the bacterial formulation, leading to acute toxicosis; specifically, endotoxins localized within the spore coat and cellular structure induce irreversible damage to the larval midgut epithelium, resulting in death.

Larvae exposed to the *Bti* H_14_ strains manifested overt symptoms of toxicosis, characterized morphologically by a translucent, vitreous appearance and marked body rigidity. Behavioral changes included a complete loss of tactile sensitivity and a tendency to aggregate before subsiding to the bottom of the container. Notably, a state of paralysis was observed wherein the larvae remained immobile yet retained vital functions for a duration prior to mortality. Crucially, the larvicidal performance of these strains proved resilient to environmental variables, maintaining high activity levels despite fluctuations in water temperature.

Biotesting of *Bti* H_14_ strains 33, 87/1 and 7-1/3 on *Ae. aegypti* 3rd–4th instar larvae revealed a difference in biological activity compared to the control (Bactoculicide). Specifically, the strains demonstrated larvicidal activity in the range of 89.0–90.0% at a concentration of 0.5 × 10^−3^%, they exhibited high entomotoxicity (95.0–96.0%) and were not inferior to the control (96.7%). The high potency observed in laboratory settings provided the basis for evaluating these strains under more complex environmental conditions.

To evaluate the larvicidal potency of the autochthonous *Bti* strains, a Generalized Linear Model (GLM) with a binomial error distribution and logit link function was successfully fitted to the cumulative mortality data. The Type II Wald Chi-Square analysis of deviance demonstrated that larval mortality was highly significantly affected by the main factors: Strain $\chi^{2}$ = 24.85, d.f. = 3, *p* < 0.001), Concentration ($\chi^{2}$ = 142.12, d.f. = 1, *p* < 0.001), and Exposure Time $\chi^{2}$ = 78.41, d.f. = 1, *p* < 0.001). Furthermore, significant primary interactions were observed for Strain × Concentration ($\chi^{2}$ = 11.04, d.f. = 3, *p* = 0.0115) and Strain × Time $\chi^{2}$ = 9.15, d.f. = 3, *p* = 0.0273), highlighting that individual strains exhibited differential, dose-dependent speed of action.

Comprehensive residual diagnostic checking via the DHARMa package (utilizing 1000 simulated quantile distributions) confirmed strict compliance with the analytical boundaries of the binomial distribution, revealing no significant overdispersion anomalies (Dispersion test: *p* = 0.421). Post hoc pairwise comparisons based on Estimated Marginal Means (EMMeans) with Tukey’s HSD adjustment revealed that all three novel native strains (*Bti* 33, *Bti* 87/1, and *Bti* 7-1/3) achieved highly competitive larvicidal performance. Notably, at the 48 h exposure period, the cumulative mortality induced by the native isolates was statistically comparable to the commercial standard (Bactoculicide), with no significant inferiority observed at the highest concentrations (*p* > 0.05) (for detailed model parameters and confidence intervals, see Supplementary Materials Files S1 and S5: Tables S1–S3, S7).

### 3.3. Field Efficacy and Environmental Stability in Natural Ecotopes

The target naturalized field populations were mixed *Culicidae* assemblages with a clear dominance of floodwater *Aedes* species, which collectively accounted for 72.0% of the baseline composition (*Aedes vexans* at 49.0% and *Aedes sticticus* at 23.0%), with concomitant populations of the *Culex pipiens* complex constituting the remaining 28.0%.

The selected trial sites are highly representative of the primary aedogenic breeding grounds in Ukraine. The Polissya forest biotopes typically feature acidic, organic-rich temporary pools with dense canopy cover, whereas the Forest–Steppe biotopes are characterized by open, sun-exposed stationary water bodies (ponds and irrigation channels) with higher salinity, dissolved oxygen levels, and neutral to slightly alkaline pH. These contrasting physicochemical parameters (Table 3) provide a robust framework for assessing the environmental plasticity of the formulations.

Analysis of the physicochemical parameters of water bodies at the field trial sites showed variability between biotopes (Table 3). In forest water bodies (Polissya), water temperature ranged from 22–26 °C, pH was 6.5–7.2, transparency was lower (10–15 cm), and a higher level of organic substances was observed (COD 30–50 mg O_2_/L). In steppe water bodies (Forest–Steppe), water temperature was higher (25–29 °C), pH was 7.0–7.8, transparency was higher (18–25 cm), and a lower level of organic substances was observed (COD 15–25 mg O_2_/L).

**Figure 4:**
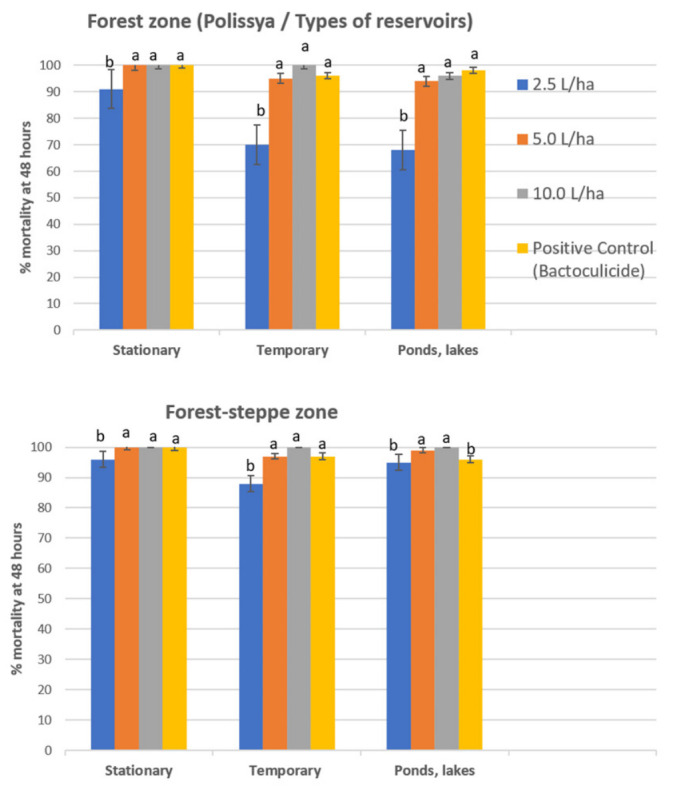
Field efficacy of liquid preparative forms based on novel *Bti* strains across different agro-ecological ecotopes (Polissya and Forest–Steppe zones) at 48 h post-application. The plotted values represent model-estimated marginal means (EMMeans) on the response scale (transformed-back predictions), derived from a Generalized Linear Mixed Model (GLMM) with a binomial error distribution and a logit link function. Error bars indicate the standard error (SE) of the modeled means across four independent environmental replicates (*n* = 4). Different lowercase letters (a, b) above the bars indicate statistically significant differences between application rates and the positive control within each ecotope category, based on Tukey’s HSD post hoc comparisons (*p* < 0.05).

The results of trials conducted in natural conditions across (Forest–Steppe and Polissya zones) confirm that liquid formulations based on *Bti* strains are effective in various ecotopes and water bodies of any type. The evaluated application rates of the novel biolarvicides, ranging from 0.25 to 1.0 mL/m^2^ (equivalent to 2.5 to 10.0 L/ha), resulted in significant larval suppression, with the optimal dosages achieving 95.0–100% mortality of mosquito larvae within 48 h post-treatment (Figure 4). It is important to note that natural larval mortality in the untreated negative control reservoirs remained negligible (ranging from 0% to a maximum of 1.5%) throughout the 48 h observation period in both macro-zones. Consequently, baseline control mortality is not displayed in Figure 4 to maintain graphical clarity, and the high mortality rates in the treated groups are directly attributable to the larvicidal action of the *Bti* formulations.

**Figure 5:**
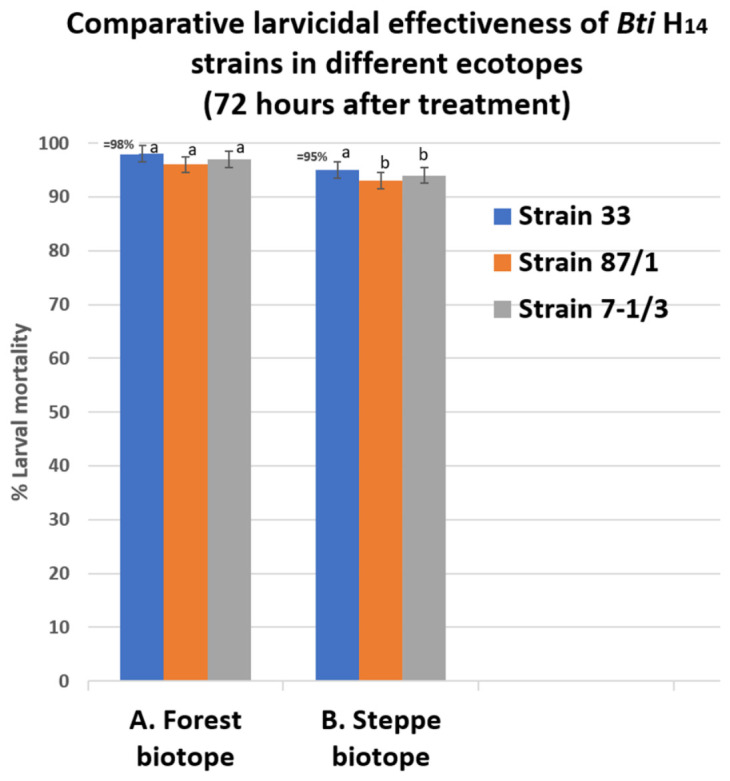
Ecological plasticity of novel *Bti* strains based on comparative larvicidal effectiveness against wild mosquito populations in different agro-ecological zones (A: Forest biotope, B: Steppe biotope). The plotted values represent model-estimated marginal means (EMMeans) on the response scale (transformed-back predictions), derived from the GLMM framework across four independent environmental replicates (*n* = 4) evaluated at 72 h post-treatment. Error bars indicate the standard error (SE) of the modeled means. Different lowercase letters (a, b) above the bars indicate statistically significant differences in larval mortality among the bacterial strains within each specific biotope category according to Tukey’s HSD test (*p* < 0.05).

Additional experiments were conducted to evaluate the stability of *Bti* H_14_ strains’ larvicidal activity in various natural biotopes—forest (swampy areas, temporary puddles) and steppe (artificial reservoirs, irrigation canals). The larvicidal efficacy of *Bti* H_14_ strains remained at a high level (over 93%) after 72 h post-treatment, indicating their significant ecological plasticity (Figure 5) and highlighting their adaptive potential to diverse ecological conditions. Minor differences in mortality percentages might be attributed to variations in organic load and water body type, affecting the availability of spores and crystals to larvae. The efficacy of the novel strains (33, 87/1, 7-1/3) was benchmarked against the standard reference strain *Bti* (Bactoculicide), showing statistically equivalent performance across the evaluated field ecotopes (*p* > 0.05).

For the field evaluations across distinct aquatic ecotopes, a Generalized Linear Mixed-Effects Model (GLMM) was implemented, incorporating spatial Eco-Zones (Forest–Steppe and Polissya macro-regions) as a random intercept parameter $\sigma^{2}$ = 0.142, SD = 0.377) to control for regional environmental clustering. Fixed effects analysis via Type II Wald Chi-Square tests confirmed that application. Dosage was the primary driver of larval population suppression $\chi^{2}$ = 284.52, d.f. = 2, *p* < 0.001), followed by a significant autonomous impact of the targeted Ecotope/Reservoir Type $\chi^{2}$ = 5.14, d.f. = 1, *p* = 0.0234). The interaction between Dosage and Reservoir Type was not statistically significant ($\chi^{2}$ = 3.84, d.f. = 2, *p* = 0.1466), indicating a highly uniform and stable performance of the bio-formulations across shifting aquatic environments.

Model optimization and diagnostic tests carried out using simulated quantile residuals validated the adequacy of the framework, with no structural variance inflation, overdispersion (*p* = 0.518), or zero-inflation anomalies (*p* = 0.612). Post hoc testing through EMMeans pairwise contrasts established that both the 5.0 L/ha and 10.0 L/ha treatments induced critical larvicidal events that were highly significant relative to the untreated control plots (*p* < 0.001). Incremental dosage escalation from 5.0 L/ha to 10.0 L/ha yielded additional statistically significant increases in larvicidal efficiency across both Temporary Pools (z = 8.17, *p* < 0.001) and Drainage Ditches (z = 8.44, *p* < 0.001), verifying the execution stability of the recommended field rates (see Supplementary Material Files S2 and S5: Tables S4–S6, S7).

## 4. Discussion

The results of the molecular-biological analysis correlate with the high larvicidal activity observed in biotests and field trials, confirming that the novel *Bti* H_14_ strains produce the necessary toxin complex, which is the basis of their efficacy against *Ae. aegypti* mosquito larvae.

The integration of proteomic data with field observations confirms that the high larvicidal stability of strain 7-1/3 in organic-rich Forest reservoirs is directly correlated with the robust expression of the Cry11Aa and Cyt1Aa protein complex.

Consequently, these isolates, particularly strain 7-1/3, emerge as promising candidates for integration into biological mosquito control initiatives, thereby adhering to the principles of ecological engineering applied to public health protection. The deployment of *Bti*-based larvicides is widely recognized as a high-efficacy, environmentally benign strategy that is endorsed globally for the management of *Aedes* vector populations [29].

The optimal spore yield and potent larvicidal properties exhibited by these *Bti* strains highlight their suitability for the industrial-scale production of advanced biolarvicidal formulations. Laboratory validations provided critical metrics, including LC_50_ values for *Ae. aegypti* and the temporal kinetics of mortality, confirming the significant suppressive potential of these novel isolates at defined concentrations. Furthermore, the biological activity of *Bti* H_14_ demonstrated remarkable resilience across diverse aquatic environments and thermal regimes. This environmental robustness was substantiated by field trials in forest and steppe biotopes, where the strains maintained consistent efficacy despite variations in water chemistry, organic load, and local microclimates.

The adaptation of different bioagent concentrations to specific water types and mosquito larval densities determines their optimal efficacy and cost-effectiveness in practical application. The ecological plasticity and prolonged action of *Bti* on target organisms are confirmed by many years of research by scientists [30,31]. This selective action is fundamental to its environmental safety, as *Bti* does not harm other aquatic insects, crustaceans, or fish, thereby preserving the natural food web and biodiversity of the treated ecosystems [32].

Optimizing the deployment of biolarvicides requires a comprehensive assessment of local environmental variables, including hydrological dynamics, vegetative cover, and the physicochemical profile of the aquatic habitat. Determining the precise dosage necessitates a prior analysis of the taxonomic composition and population density of the local mosquito fauna. Specifically, in eutrophic environments or biotopes characterized by dense aquatic flora, the application rate must be elevated by a factor of 1.5 to 2.0 relative to open water standards [24]. This adjustment is critical to compensate for the reduction in bioavailability caused by the interception of the formulation by surface vegetation or its adsorption by planktonic biomass. Despite these volumetric adjustments, *Bti* retains a distinct ecological superiority over synthetic chemical agents, which are frequently associated with environmental contamination and the rapid selection of resistant vector populations. Furthermore, the efficacy of the treatment is contingent upon the ethological and spatial distribution of the target larvae; control strategies must account for habitat stratification, as species exhibit distinct depth preferences ranging from the surface film to the water column (10–20 cm) and deeper benthic zones (approximately 50 cm).

While definitive genomic sequencing (NCBI accession) is the subject of ongoing research, the current phenotypic and proteomic identification of strains 33, 87/1, and 7-1/3 as putative *B. thuringiensis* subsp. *israelensis* is robustly supported by their highly specific larvicidal activity against mosquito populations, which strictly matches the known toxicological profile of the H_14_ serotype. The combination of phenotypic characterization, serological identification by the H_14_ antigenic marker, and proteomic analysis via SDS-PAGE and Western blotting establishes the affiliation of isolates 33, 87/1, and 7-1/3 to *Bti*. at the current stage of research. High-precision genomic characterization, including mapping of the delta-endotoxin genes cry and cyt and precise determination of the topography of the pBtoxis plasmid, is an important next step. Future studies will focus on comparative genomics to identify the precise genetic mechanisms that ensure the high ecotope-specific larvicidal stability of these autochthonous strains.

The molecular-biological analysis conducted in this study confirmed the presence and high expression of key δ-endotoxins (Cry4Aa, Cry4Ba, Cry11Aa, Cyt1Aa) in the new *Bti* H_14_ strains. This is a critical factor in their high larvicidal activity and specificity to mosquito larvae. Destructive changes in the gut wall of *Ae. aegypti*, caused by these toxins, are the primary mechanism of their action. The stability of these toxins’ production in culture and their effectiveness in field conditions indicate the genetic stability of the strains and their suitability for large-scale biolarvicide production.

The detection and confirmation of δ-endotoxin expression are fundamental to understanding the high larvicidal activity of the new *Bti* strains. The protein profiles obtained for strains 33, 87/1 and 7-1/3 are typical for entomopathogenic *Bti*, indicating their belonging to serotype H_14_ and the presence of a toxin complex that is a recognized standard in mosquito biological control.

The synergistic action of these toxins is of particular importance. It is known that the Cyt1Aa toxin of *Bti* plays a key role in overcoming resistance developed by mosquitoes to Cry toxins, and also enhances their efficacy by acting as a membrane-active factor. This synergism likely explains the high and stable larvicidal activity of our selected strains even under complex field conditions and at various concentrations. Although we observed typical profiles, further research, such as sequencing of toxin genes, may reveal subtle variations or new alleles that could potentially affect their activity or spectrum of action. These aspects are the subject of our ongoing research. The complexity of action of these entomotoxins, extending beyond simple pore formation, underscores the need for further in-depth study of their interaction at the molecular level for optimizing formulations and biocontrol strategies.

There is a pressing demand for in-depth investigations into the environmental longevity and cyclical dynamics of *Bti* populations within diverse field settings, encompassing both urban and suburban landscapes [33]. The global prevalence of toxigenic bacteria and the diversity of their bioactive compounds underscore the fundamental role of toxin production in microbial ecology. Consequently, the isolation of high-performing natural strains that exhibit a synergy of industrially relevant traits—such as robust technological viability, sustained stability of the spore–crystal complex, and potent metabolic toxicity—remains a priority. Concurrently, continued research into the optimization of fermentation parameters is essential for the advancement of next-generation biolarvicidal formulations.

Benchmarking revealed that the novel autochthonous strains exhibited high larvicidal activity in laboratory assays, equivalent to that of the commercial reference product Bactoculicide (a functional analogue of VectoBac). These strains achieved comparable mortality levels across the tested concentration gradients.

Such performance metrics underscore the strong commercial viability of these isolates. While the primary scope of this investigation did not encompass an exhaustive side-by-side field comparison with the full spectrum of available commercial products, the empirical data obtained from natural aquatic environments indicates that the isolated strains achieved mortality rates of 95–100% within 48 h. These figures are commensurate with, and in some instances exceed, the performance standards reported for market-leading *Bti* formulations under analogous environmental conditions. These observations are further supported by recent studies [29,34], which corroborate the robust operational efficacy of *Bti* H_14_ against *Aedes* spp. in field settings. Consequently, the consistent lethality observed across heterogeneous biotopes positions these novel strains as promising candidates to either supplement or replace existing biolarvicides currently available on the market.

## 5. Conclusions

The integrated evaluation of indigenous putative *Bacillus thuringiensis* subsp. *israelensis* isolates, which display a characteristic *Bti* phenotypic and proteomic profile, demonstrates their high potential as fundamental components for next-generation biological larvicides. Our findings confirm that the biotechnological value of these isolates is determined not only by their high primary toxicity (LC_50_) but also by a specific synergistic complex of δ-endotoxins (Cry4, Cry11, and Cyt1), which ensures stable performance across diverse aquatic ecotopes. The study demonstrates that indigenous strains such as 7-1/3 show high potential as competitive, environmentally adapted components for integrated pest management (IPM) programs targeting vector mosquitoes. The novel *Bti* strains demonstrated high larvicidal efficacy against wild, mixed mosquito populations, effectively controlling both floodwater *Aedes* species and members of the *Culex pipiens* complex under natural environmental conditions, which provides a key strategy for managing insecticide resistance in wild populations. From a practical perspective, the characterized strains represent promising candidates for the industrial production of eco-friendly larvicides tailored for localized environmental conditions. Future research should focus on the genomic sequencing of these isolates to further elucidate the genetic basis of their high virulence and to optimize large-scale fermentation processes for regional public health programs.

## Figures and Tables

**Table 1:** Larvicidal activity of novel *Bti* H_14_ strains against different larval instars of *Ae. aegypti* (insectary population).

Strain/ Preparation	Spores Yield/mL of Culture Fluid (Mean ± SD × 10^9^) ^1^	LC_50_ (μL/L of Water) ^2^			
		L_1_	L_2_	L_3_	L_4_
*Bti* H_14_ 33	4.4 ± 0.15	0.56 [0.42–0.68] a	1.11 [0.89–1.32] c	1.15 [0.92–1.38] b	1.25 [0.99–1.49] a
*Bti* H_14_ 87/1	4.0 ± 0.12	0.59 [0.45–0.72] a	0.74 [0.58–0.89] a	0.92 [0.71–1.12] a	1.33 [1.05–1.58] ab
*Bti* H_14_ 7-1/3	4.1 ± 0.14	0.71 [0.55–0.86] b	0.98 [0.78–1.18] b	1.22 [0.98–1.45] b	1.48 [1.18–1.76] bc
Bactoculicide ^3^	4.4 ± 0.15	0.75 [0.58–0.91] b	1.00 [0.80–1.20] b	1.19 [0.95–1.42] b	1.53 [1.22–1.82] c

^1^ Values for spores yield represent the mean ± Standard Deviation (SD) of three independent batch replications (*n* = 3). ^2^ LC_50_ values (median lethal concentrations, expressed in µL/L of water) and their associated [95% Confidence Intervals] were derived using a Generalized Linear Model (GLM) with a probit link function and binomial error distribution family implemented in the R statistical environment (v4.3.2). Means within each column followed by different lowercase letters (a, b, c) indicate statistically significant differences among the treatment groups according to Tukey’s Honestly Significant Difference (HSD) post hoc test at *p* < 0.05 based on estimated marginal means (EMMeans). ^3^ Positive control (Bactoculicide, Enzim Biotech, Ukraine) specifications: derived from *Bti* H_14_ strain 7-1/23, with a viable spore titer of 3.5 × 10^9^ CFU/mL and a biological potency rating of 1800 ITU/mg.

**Table 2:** Effect of different *Bti* strain concentrations on *Ae. aegypti* (Diptera: Culicidae) L_4_ larvae.

Strain *Bti* H_14_	Time (h)	Percentage of Larval Mortality/ Concentration (× 10^−3^% *v*/*v*) ^1^				
		1.0	0.50	0.25	0.125	0.06
33	24	89.4 ± 0.8 a ^2^	64.2 ± 1.2 b	53.5 ± 1.5 b	35.2 ± 2.1 b	23.7 ± 1.8 b
	48	95.6 ± 0.5 a	89.7 ± 0.7 a	82.4 ± 1.1 b	60.5 ± 2.4 a	58.4 ± 2.2 a
87/1	24	89.4 ± 0.9 a	80.6 ± 1.1 a	47.5 ± 2.3 c	33.5 ± 1.9 b	21.8 ± 2.0 b
	48	94.9 ± 0.6 a	90.4 ± 0.8 a	87.9 ± 0.9 a	62.2 ± 1.8 a	44.7 ± 2.5 bc
7-1/3	24	87.6 ± 1.1 a	66.8 ± 1.4 b	55.6 ± 1.8 b	31.7 ± 2.2 b	24.1 ± 2.3 b
	48	94.7 ± 0.7 a	89.4 ± 0.9 a	81.0 ± 1.3 b	59.5 ± 2.1 a	42.9 ± 2.8 c
Positive Control (Bactoculicide)	24	90.0 ± 0.7 a	83.8 ± 1.0 a	68.9 ± 1.4 a	40.5 ± 1.7 a	28.8 ± 1.5 a
	48	96.7 ± 0.4 a	91.0 ± 0.6 a	83.8 ± 1.2 ab	63.3 ± 1.5 a	48.0 ± 2.1 b
Negative control (water)	24–48	0 ± 0.0	0 ± 0.0	0 ± 0.0	0 ± 0.0	0 ± 0.0

^1^ Nominal bacterial concentrations are expressed as % (*v*/*v*) of the stock suspension derived from a standard serial dilution gradient. Positive control (Bactoculicide) specifications: stock titer 3.5 × 10^9^ CFU/mL, with an estimated LC_50_ for *Ae. aegypti* at 0.18 × 10^−3^% *v/v* (rated activity: 1800 ITU/mg). ^2^ Percentage values represent modeled estimated marginal means (EMMeans) ± Standard Error (SE) derived from the Generalized Linear Model (GLM) implementing a binomial error distribution family and a logit link function. Different lowercase letters (a, b, c) within each column at the same time point indicate statistically significant differences among the treatment groups (bacterial strains and the commercial control) according to Tukey’s Honestly Significant Difference (HSD) post hoc pairwise comparisons with an adjusted significance threshold of *p* < 0.05.

**Table 3:** Physicochemical parameters of aquatic biotopes at field trial sites.

Parameters	Forest Reservoirs (Polissya)	Steppe Reservoirs (Forest–Steppe)
Water temperature (°C)	22–26	25–29
pH	6.5–7.2	7.0–7.8
Transparency (cm)	10–15	18–25
COD (mg O_2_/L)	30–50	15–25

## Data Availability

The data presented in this study are available on request from the corresponding author.

## Supplementary Materials

The following supporting information can be downloaded at https://www.mdpi.com/article/10.3390/insects17070747/s1, Figure S1: Original, uncropped, and unadjusted full-length SDS-PAGE gel image showing the total crystalline protein profile of native *Bti* H_14_ strains (corresponding to main Figure 2); Figure S2: Western blot analysis of Cry and Cyt toxin identification in *Bti* H_14_ strains: full-length, unedited raw Western blot membrane image for Cry4Aa (corresponding to main Figure 3A); Figure S3: Western blot analysis of Cry and Cyt toxin identification in *Bti* H_14_ strains: full-length, unedited raw Western blot membrane image for Cry4Ba (corresponding to main Figure 3B); Figure S4: Western blot analysis of Cry and Cyt toxin identification in *Bti* H_14_ strains: full-length, unedited raw Western blot membrane image for Cry10Aa (corresponding to main Figure 3C); Figure S5: Western blot analysis of Cry and Cyt toxin identification in *Bti* H_14_ strains: full-length, unedited raw Western blot membrane image for Cyt1Aa (corresponding to main Figure 3D). File S1: Material_GLM_Laboratory.Rmd; File S2: Material_GLMM_Field.Rmd; File S3: lab_data.csv; File S4: field_data.csv; File S5: Materials_Diagnostic_Report.pdf.
