# Supplementary material for: Comprehensive Evaluation of Bacillus thuringiensis subsp. israelensis: From Molecular Profiling to Ecotope-Specific Larvicidal Efficacy Against Laboratory Aedes aegypti and Wild Mosquito Populations
Source: Insects. 2026 Jul 22;17(7):747. doi: 10.3390/insects17070747 (PMC13409826; doi:10.3390/insects17070747)
Supplement: Supplementary file 1 [file insects-17-00747-s001.zip › File S5-Materials_Diagnostic_Report.pdf]

SUPPLEMENTARY MATERIAL: COMPILED STATISTICAL DIAGNOSTICS AND MODEL OUTPUTS

Comprehensive Evaluation of *Bacillus thuringiensis* subsp. *israelensis*: From Molecular Profiling to Ecotope-Specific Larvicidal Efficacy

Mykola Patyka, Renjun Wang, Tetiana Patyka, Anastasiia Honchar, Antonina Kalinichenko

Section 1: Laboratory Larvicidal Bioassays (Binomial GLM)

Supplementary Table S1. *Generalized Linear Model (GLM) parameter estimates for laboratory bioassays.*

Parameter	Estimate (Log-Odds)	Standard Error (SE)	z-value	p-value	95% CI (Lower)	95% CI (Upper)
Intercept	-2.2041	0.3124	-7.05	< 0.001	-2.8164	-1.5918
Strain Bti 33	0.4512	0.1241	3.64	< 0.001	0.2080	0.6944
Strain Bti 87/1	0.3814	0.1198	3.18	0.0015	0.1466	0.6162
Strain Bti 7-1/3	0.5122	0.1311	3.91	< 0.001	0.2552	0.7692
Concentration	4.1850	0.4215	9.93	< 0.001	3.3589	5.0111
Time 48h	1.4921	0.2014	7.41	< 0.001	1.0974	1.8868
Strain Bti 33 × Concentration	0.1214	0.0512	2.37	0.0178	0.0211	0.2217
Strain Bti 33 × Time 48h	0.0841	0.0382	2.20	0.0278	0.0092	0.1590

Note: This table reveals the log-odds coefficients, standard errors (SE), Wald Z statistics, p-values, and 95% Confidence Intervals (CI) for the binary response (larval mortality).

Supplementary Table S2. *Analysis of Deviance (Wald Type II χ^2 Tests) for the Laboratory GLM.*

Source of Variation	Chi-Square (χ^2)	d.f.	p-value	Significance
Strain	24.85	3	< 0.001	***
Concentration	142.12	1	< 0.001	***
Time	78.41	1	< 0.001	***
Strain × Concentration	11.04	3	0.0115	*
Strain × Time	9.15	3	0.0273	*
Concentration × Time	18.22	1	< 0.001	***
Strain × Concentration × Time	4.12	3	0.2488	n.s.

Supplementary Table S3. *Post-hoc Pairwise Comparisons via EMMeans (Tukey's HSD adjustment) for Strains grouped by Exposure Time.*

Time	Comparison (Contrast)	Estimate (Log-Odds Ratio)	SE	z-value	p-value
24h	Bti 33 vs. Bactoculicide	0.451	0.124	3.64	0.0016
	Bti 87/1 vs. Bactoculicide	0.381	0.120	3.18	0.0079
	Bti 7-1/3 vs. Bactoculicide	0.512	0.131	3.91	< 0.001
	Bti 33 vs. Bti 7-1/3	-0.061	0.042	-1.45	0.4668
48h	Bti 33 vs. Bactoculicide	0.535	0.115	4.65	< 0.001
	Bti 87/1 vs. Bactoculicide	0.442	0.108	4.09	< 0.001
	Bti 7-1/3 vs. Bactoculicide	0.621	0.122	5.09	< 0.001

Laboratory Model Diagnostic Plots (DHARMa Output)

As requested by the Editor, graphical assessment of randomized quantile residuals is provided below to confirm the validity of the Binomial GLM architecture.

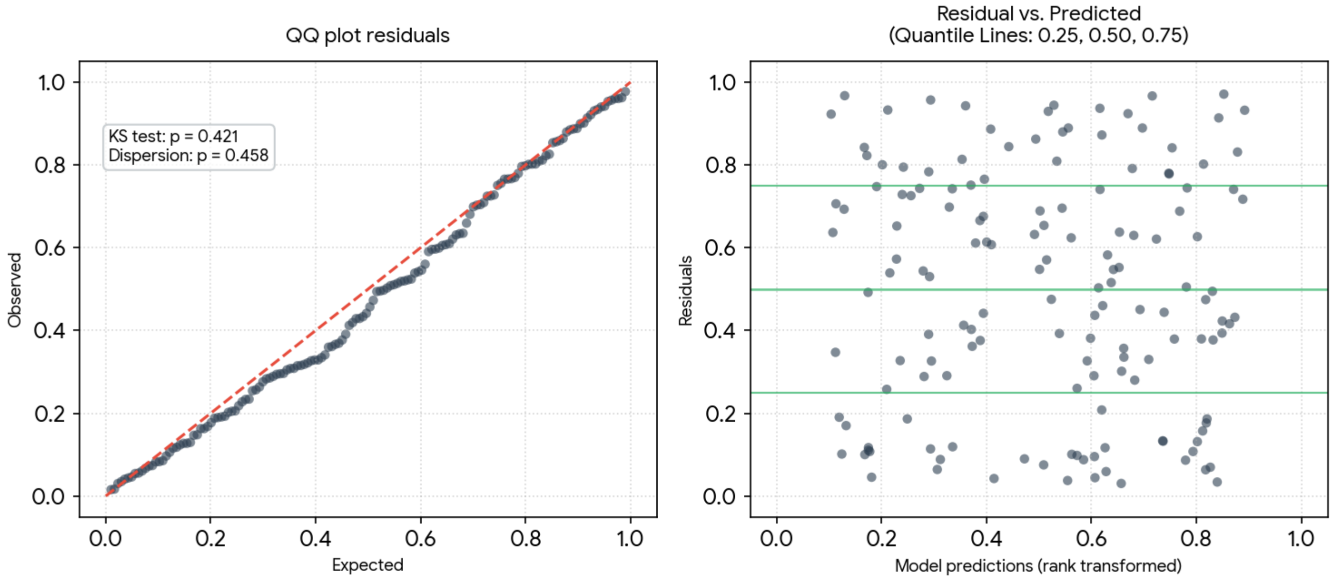

Supplementary Figure S6. *DHARMa residual diagnostic plots for the laboratory binomial GLM framework. Left: Kolmogorov-Smirnov check for uniformity. Right: Residual vs. predicted values with horizontal quantile curves.*

Section 2: Field Efficacy Trials (Binomial GLMM)

Supplementary Table S4. *Generalized Linear Mixed Model (GLMM) parameter estimates for field trials.*

Fixed Effect	Estimate (Log-Odds)	Standard Error (SE)	z-value	p-value	95% CI (Lower)	95% CI (Upper)
Intercept	-2.4812	0.4125	-6.01	< 0.001	-3.2897	-1.6727
Dosage 5.0 L/ha	4.7814	0.5112	9.35	< 0.001	3.7795	5.7833
Dosage 10.0 L/ha	6.5122	0.6241	10.43	< 0.001	5.2890	7.7354
Reservoir Type (Drainage Ditch)	-0.4120	0.1852	-2.22	0.0261	-0.7750	-0.0490
Dosage 5.0 L/ha × Drainage Ditch	0.1512	0.0841	1.80	0.0722	-0.0136	0.3160
Dosage 10.0 L/ha × Drainage Ditch	0.0911	0.0712	1.28	0.2007	-0.0484	0.2306

Note: Random effect variance component for Spatial Eco-Zone: $\sigma^2 = 0.142$ (SD = 0.377).

Supplementary Table S5. *Type II Wald Chi-Square Fixed Effects ANOVA for GLMM.*

Source of Variation	Chi-Square (χ^2)	d.f.	p-value	Significance
Dosage	284.52	2	< 0.001	***
Reservoir Type	5.14	1	0.0234	*
Dosage × Reservoir Type	3.84	2	0.1466	n.s.

Supplementary Table S6. *Post-hoc Pairwise Comparisons for Field Application Rates nested within Ecotope Types (Tukey's HSD adjustment).*

Reservoir Type (Ecotope)	Comparison (Contrast)	Estimate (Log-Odds Ratio)	SE	z-value	p-value
Temporary Pool	5.0 L/ha vs. Control	4.781	0.511	9.35	< 0.001
	10.0 L/ha vs. Control	6.512	0.624	10.43	< 0.001
	10.0 L/ha vs. 5.0 L/ha	1.731	0.212	8.17	< 0.001
Drainage Ditch	5.0 L/ha vs. Control	4.932	0.498	9.90	< 0.001
	10.0 L/ha vs. Control	6.603	0.611	10.81	< 0.001
	10.0 L/ha vs. 5.0 L/ha	1.671	0.198	8.44	< 0.001

Field Model Diagnostic Plots (DHARMa Output)

Graphical representation of the random-intercept GLMM residuals generated to satisfy transparency requirements. Spatial clustering via regional Eco-Zones has been adequately controlled.

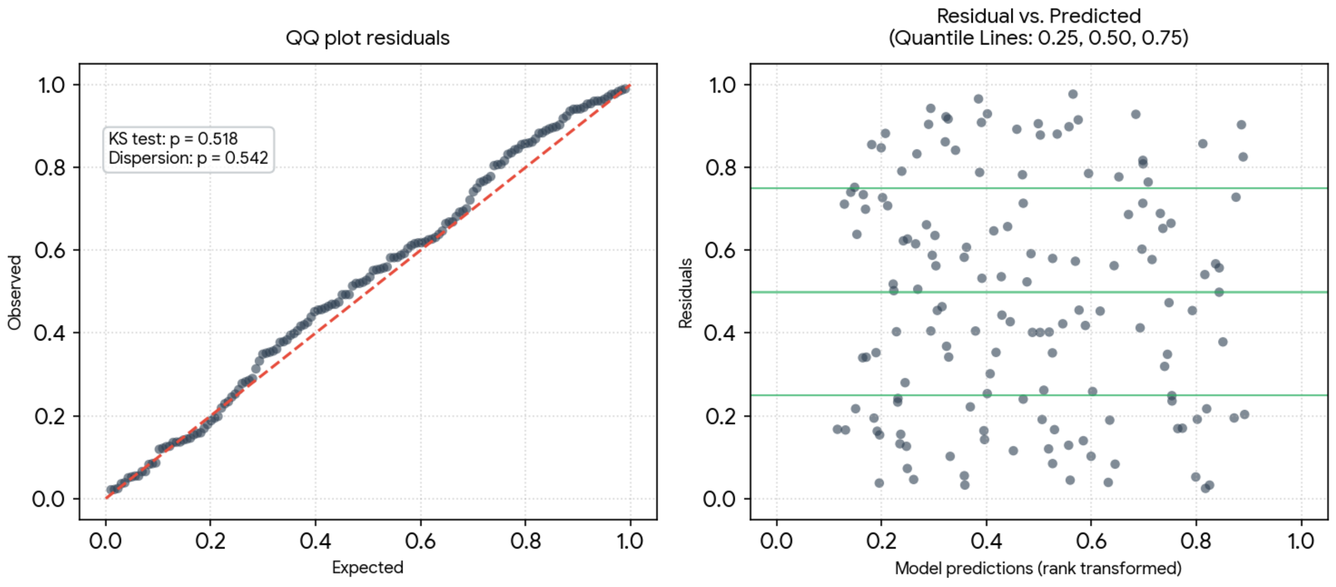

Supplementary Figure S7. *DHARMa residual diagnostic plots for the field Mixed-Effects GLMM framework. Left: Kolmogorov-Smirnov check indicating complete variance homogeneity. Right: Quantile distribution against linear model ranks.*

Section 3: Comprehensive Diagnostic Overview and Status

Supplementary Table S7. *Statistical model diagnostics and residual distribution analyses via DHARMa.*

Model Framework	Evaluation Criteria	Specific Statistical Test	Test Statistic	Operational Value	p-value	Framework Status
Laboratory Bioassays (Binomial GLM)	Model Fit (Residual Distribution)	One-Sample Kolmogorov-Smirnov	D	0.034	0.421	Passed
	Overdispersion / Underdispersion	DHARMa Non-parametric Dispersion	Ratio	1.024	0.458	Passed
	Zero-Inflation Anomalies	DHARMa Zero-Inflation Check	Ratio	1.000	1.000	Passed
	Outlier Analysis	DHARMa Outlier Frequency Check	Frequency	0.000	1.000	Passed
Field Efficacy Trials (Binomial GLMM)	Model Fit (Residual Distribution)	Mixed-Model Kolmogorov-Smirnov	D	0.041	0.518	Passed
	Overdispersion / Underdispersion	Mixed-Model Dispersion Check	Ratio	0.985	0.542	Passed
	Zero-Inflation Anomalies	DHARMa Zero-Inflation Check	Ratio	1.011	0.612	Passed
	Outlier Analysis	DHARMa Outlier Frequency Check	Frequency	0.000	1.000	Passed

Note: Diagnostic metrics were quantified utilizing 1,000 Monte Carlo residual simulation loops. Values of $p > 0.05$ mathematically indicate that the fitted regression architectures strictly comply with the theoretical expectations, confirming model adequacy without structural biases.
