# Supplementary figures and images for: Comprehensive Evaluation of Bacillus thuringiensis subsp. israelensis: From Molecular Profiling to Ecotope-Specific Larvicidal Efficacy Against Laboratory Aedes aegypti and Wild Mosquito Populations
Source: Insects. 2026 Jul 22;17(7):747. doi: 10.3390/insects17070747 (PMC13409826; doi:10.3390/insects17070747)

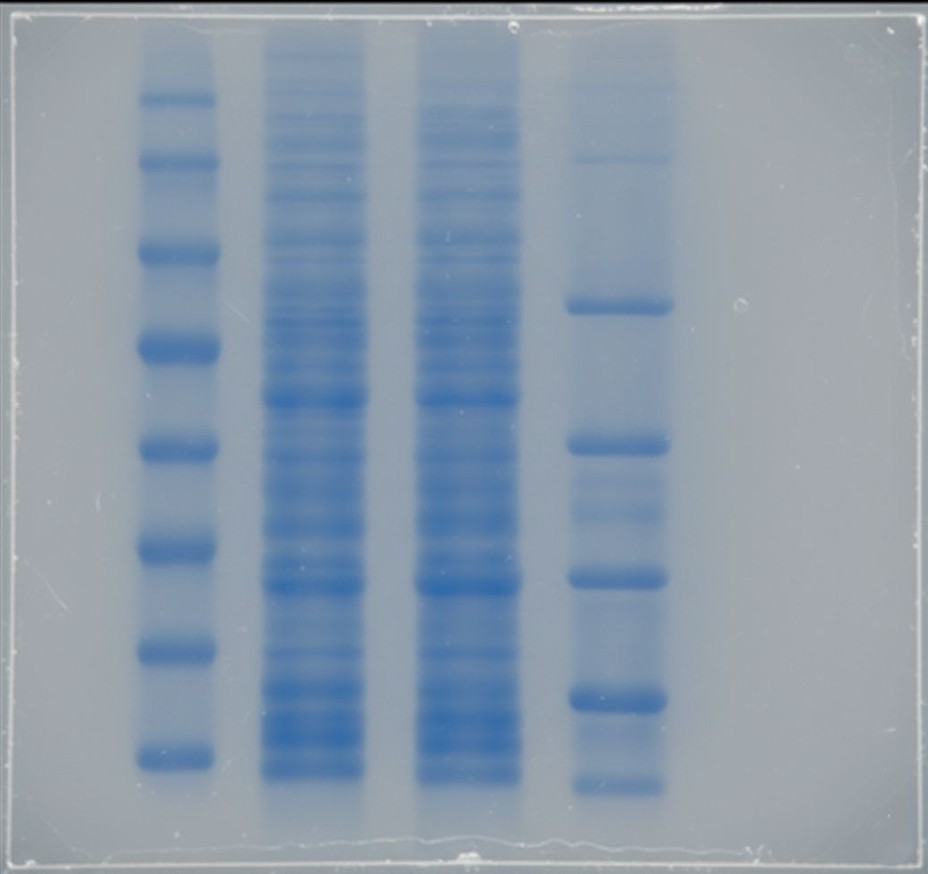

Supplement: Supplementary file 1 [file insects-17-00747-s001.zip › Figure S1.jpg]

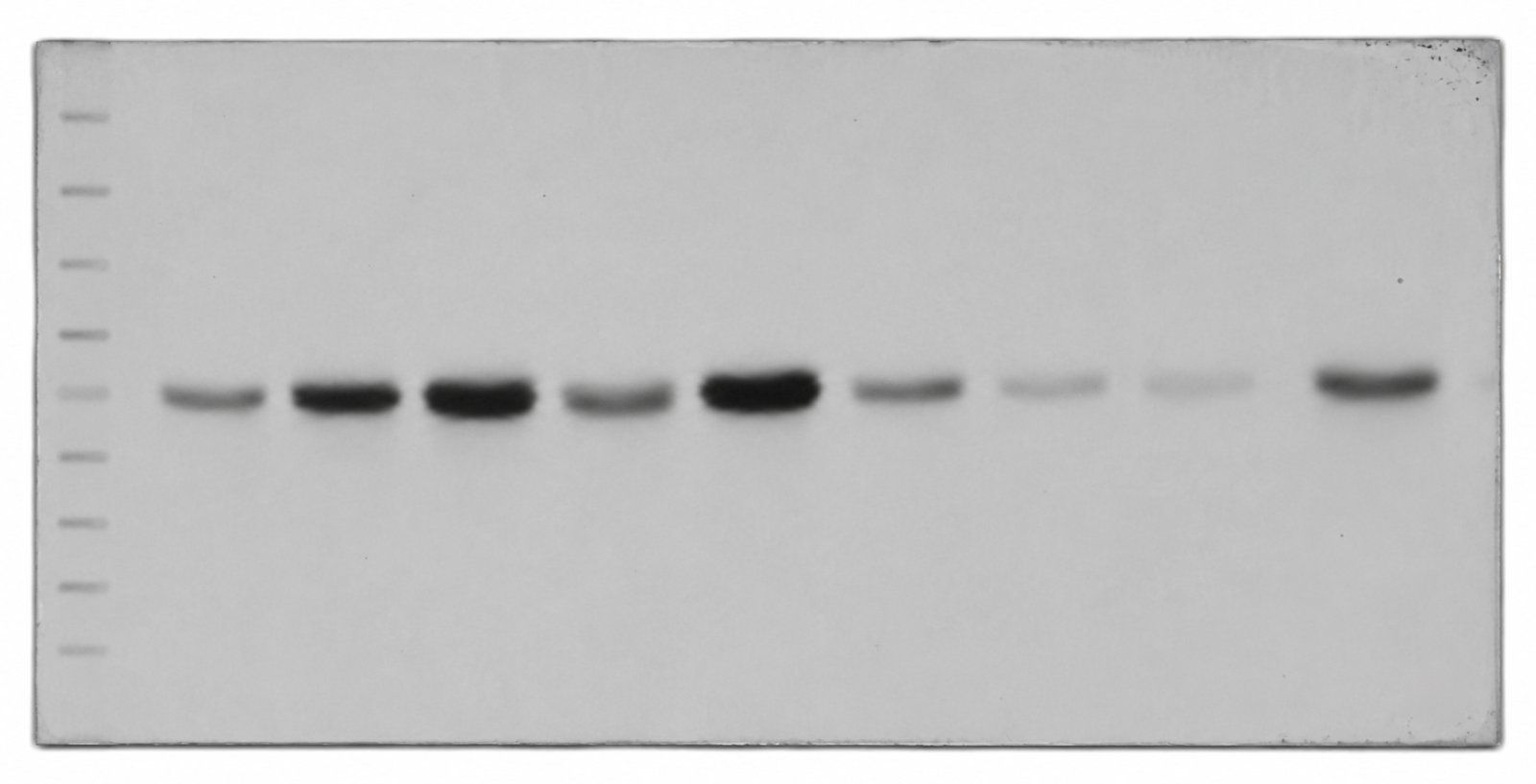

Supplement: Supplementary file 1 [file insects-17-00747-s001.zip › Figure S2.jpg]

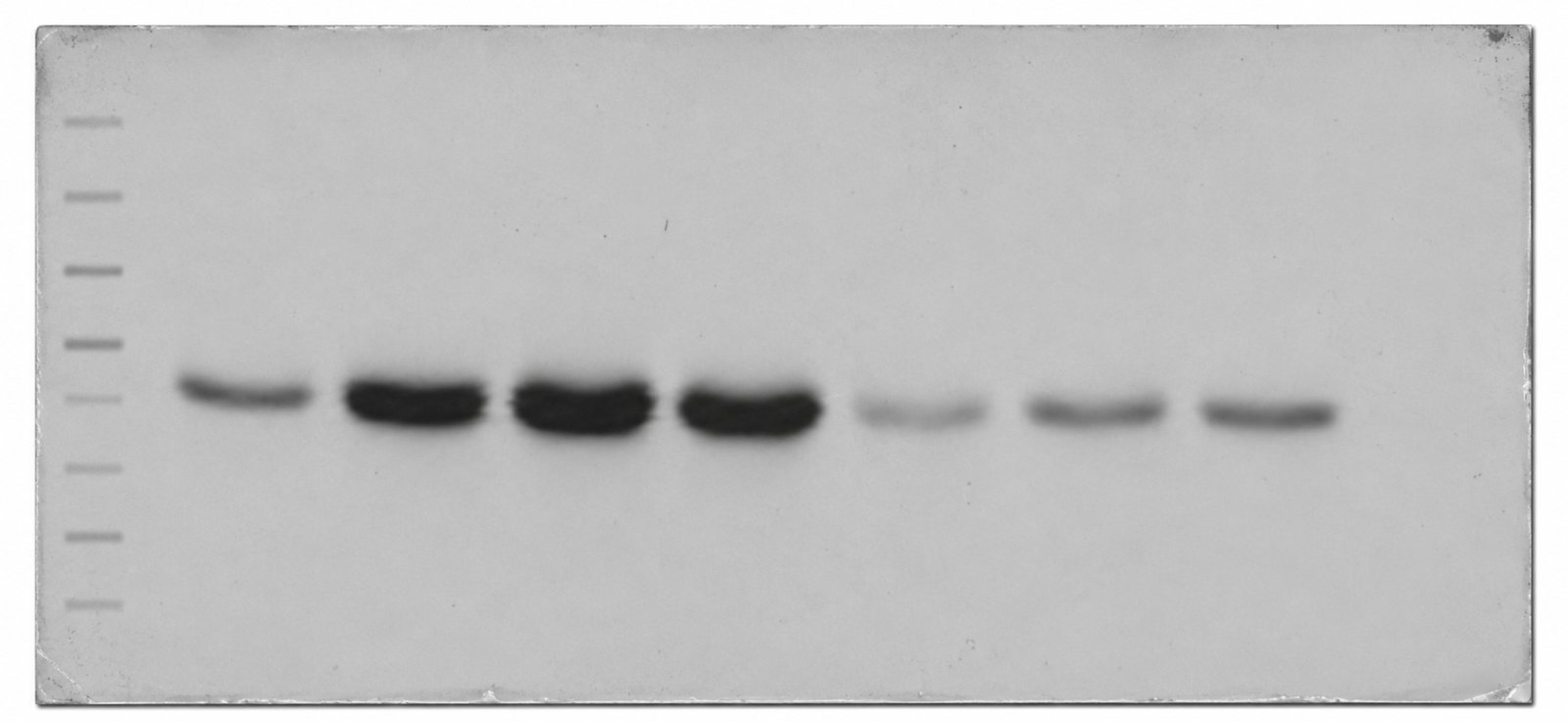

Supplement: Supplementary file 1 [file insects-17-00747-s001.zip › Figure S3.jpg]

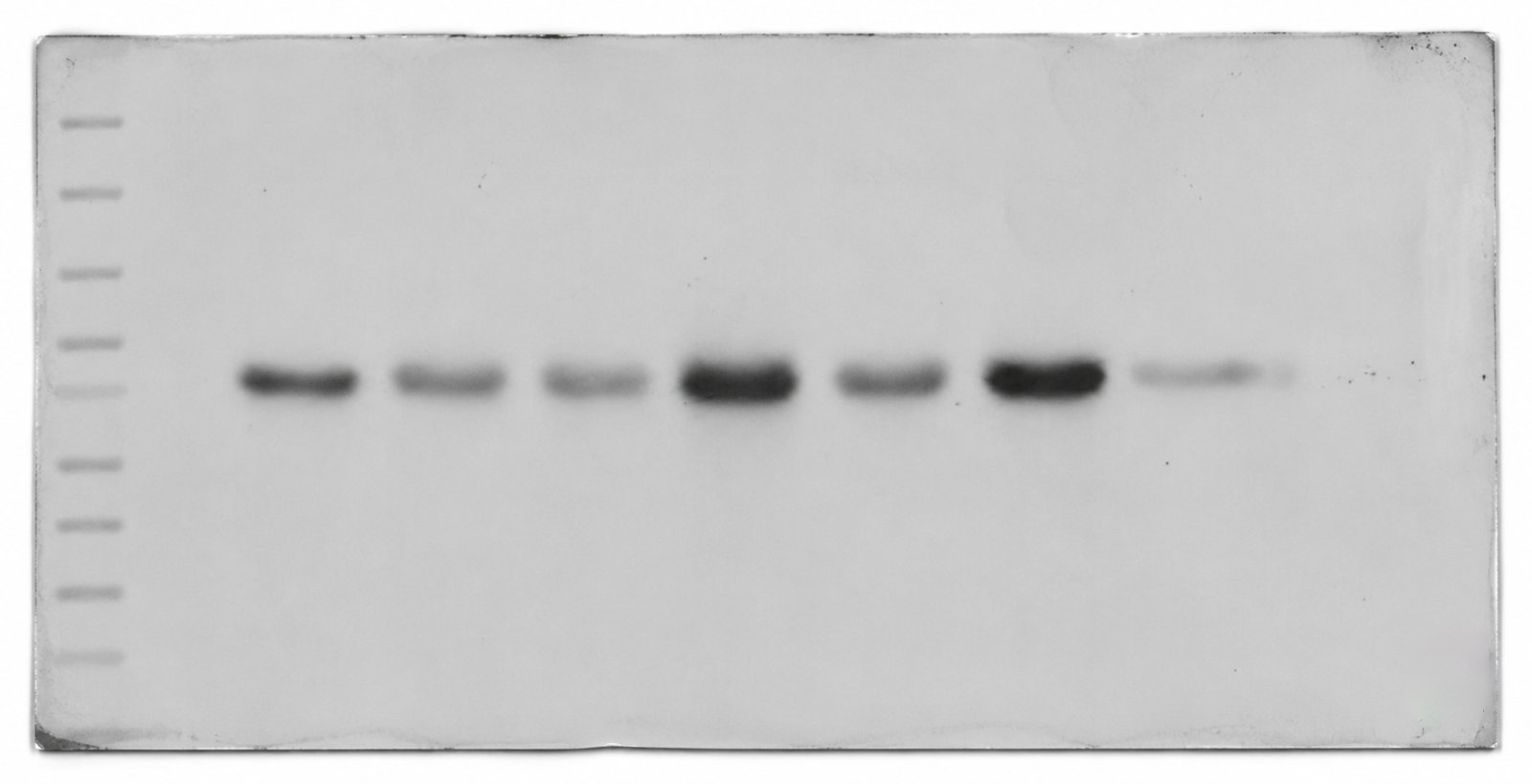

Supplement: Supplementary file 1 [file insects-17-00747-s001.zip › Figure S4.jpg]

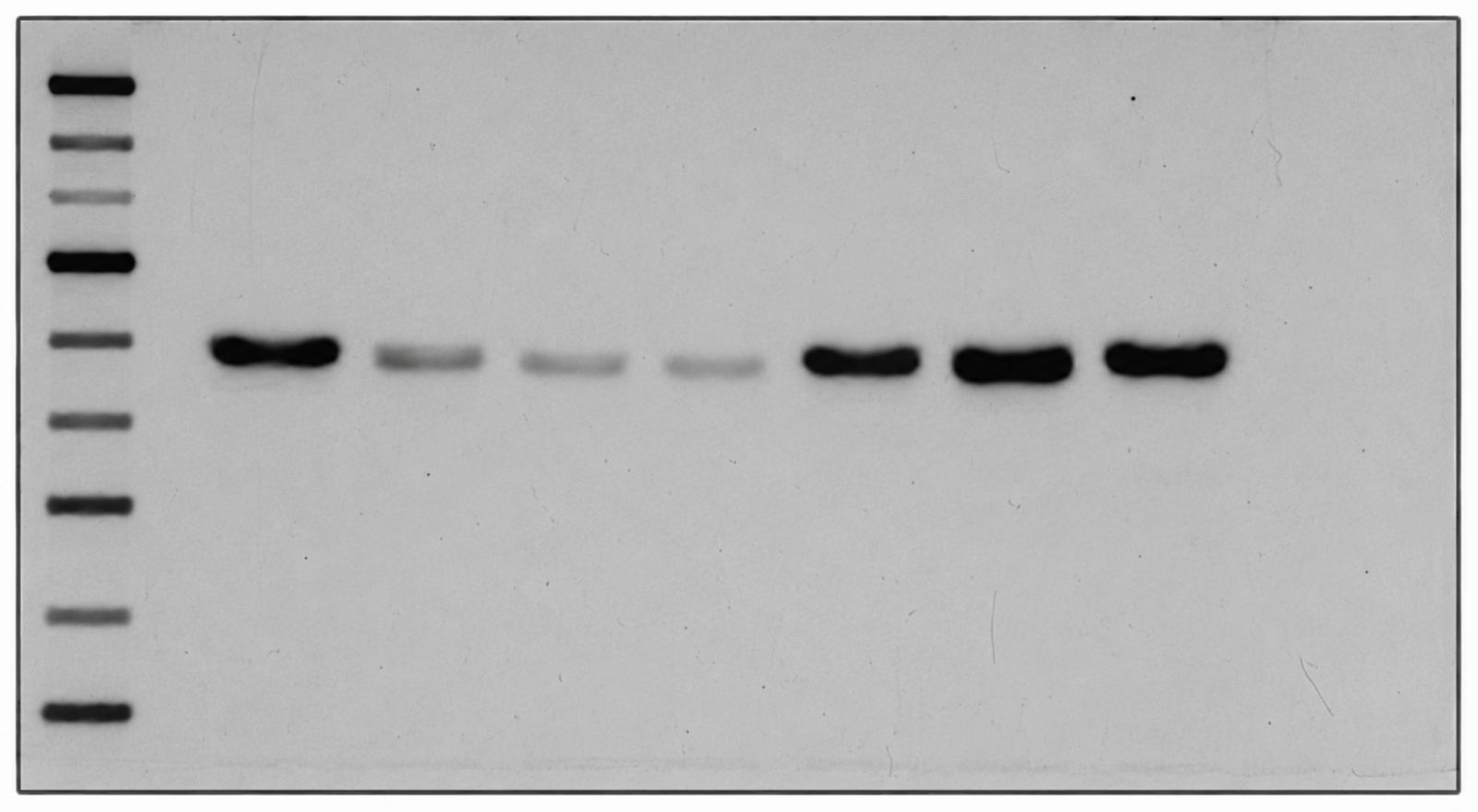

Supplement: Supplementary file 1 [file insects-17-00747-s001.zip › Figure S5.jpg]
